# An examination of targeted gene neighborhoods in strawberry

**DOI:** 10.1186/1471-2229-10-81

**Published:** 2010-05-04

**Authors:** Thomas M Davis, Melanie E Shields, Qian Zhang, Denise Tombolato-Terzić, Jeffrey L Bennetzen, Ana C Pontaroli, Hao Wang, Qin Yao, Phillip SanMiguel, Kevin M Folta

**Affiliations:** 1 Department of Biological Sciences, University of New Hampshire, Durham, NH 03824 USA; 2 Department of Molecular, Cellular & Biomedical Sciences, University of New Hampshire, Durham, NH 03824 USA; 3 Department of Genetics, University of Georgia, Athens, GA 30602 USA; 4 Department of Horticulture and Landscape Architecture, Purdue Univ., West Lafayette, IN 47907 USA; 5 Horticultural Sciences Department and Plant Molecular and Cellular Biology Program, PO Box 110690, 1301 Fifield Hall, Gainesville, FL 32611 USA; 6 Estación Experimental Agropecuaria Balcarce, Instituto Nacional de Tecnología Agropecuaria (INTA) - Consejo Nacional de Investigaciones Científicas y Técnicas (CONICET); CC 276 (7620) Balcarce, Argentina

## Abstract

**Background:**

Strawberry (*Fragaria *spp.) is the familiar name of a group of economically important crop plants and wild relatives that also represent an emerging system for the study of gene and genome evolution. Its small stature, rapid seed-to-seed cycle, transformability and miniscule basic genome make strawberry an attractive system to study processes related to plant physiology, development and crop production; yet it lacks substantial genomics-level resources. This report addresses this deficiency by characterizing 0.71 Mbp of gene space from a diploid species (*F. vesca*). The twenty large genomic tracks (30-52 kb) captured as fosmid inserts comprise gene regions with roles in flowering, disease resistance, and metabolism.

**Results:**

A detailed description of the studied regions reveals 131 Blastx-supported gene sites and eight additional EST-supported gene sites. Only 15 genes have complete EST coverage, enabling gene modelling, while 76 lack EST support. Instances of microcolinearity with *Arabidopsis thaliana *were identified in twelve inserts. A relatively high portion (25%) of targeted genes were found in unanticipated tandem duplications. The effectiveness of six FGENESH training models was assessed via comparisons among *ab initio *predictions and homology-based gene and start/stop codon identifications. Fourteen transposable-element-related sequences and 158 simple sequence repeat loci were delineated.

**Conclusions:**

This report details the structure and content of targeted regions of the strawberry genome. The data indicate that the strawberry genome is gene-dense, with an average of one protein-encoding gene or pseudogene per 5.9 kb. Current overall EST coverage is sparse. The unexpected gene duplications and their differential patterns of EST support suggest possible subfunctionalization or pseudogenization of these sequences. This report provides a high-resolution depiction of targeted gene neighborhoods that will aid whole-genome sequence assembly, provide valuable tools for plant breeders and advance the understanding of strawberry genome evolution.

## Background

Strawberry is the familiar name of a valuable genus [*Fragaria *spp. (Rosaceae)] comprising cultivated plants that produce popular and nutritious fruits, as well as wild plant species that populate many areas of the northern hemisphere and South America. The genus contains a diversity of species representing ploidy levels from diploid to decaploid [1,2]. The octoploid (2 n = 8 x = 56) genome composition of the cultivated strawberry, *Fragaria *× *ananassa*, places this hybrid species among the most genetically complex crop plants. Paradoxically, the ~200 Mbp size of the basic (x = 7) strawberry genome [3,4] ranks among the lowest of crop plant C values. The discordance between small basic genome size and wide ranging genomic complexities makes *Fragaria *a unique system in which to study the effects of reticulate evolution, polyploidization, domestication, and breeding on genes and genomes.

While the cultivated strawberry's genome is complex, extant diploid species provide the opportunity to analyze simpler yet relevant genomes as appropriate comparators to the cultivated octoploids. The widely distributed diploid (2 n = 2 x = 14) species *Fragaria vesca *has been implicated as an ancestral subgenome donor to the octoploid strawberries: *F*. ×*ananassa *and its immediate ancestors *F. chiloensis *and *F. virginiana *[5]. Thus, *F. vesca *provides an outstanding system for addressing genetic and genomic questions relevant to the octoploid strawberries, as well as to the entire Rosaceae family [6]. Existing genomics resources for *F. vesca *include mapping populations and linkage maps [7,8], an efficient genetic transformation system for reverse genetics [9,10], growing EST support [11], and forthcoming whole-genome sequence information [12].

Large scale projects aimed at sequencing the complete genomes of many crop and model plant species have been preceded by smaller scale efforts that informatively sample and annotate limited but representative genomic regions. Examples include the sequencing of: an initial 1.9 Mb contig from *Arabidopsis thaliana *chromosome 4 [13], constituting about 1.4% of the 140 Mbp *Arabidopsis *genome; six BAC clones, comprising 592 kb or 0.06% of the 950 Mbp tomato genome [14]; and BAC ends that generated 17 Mb of sequence or 4.7% of the 372 Mbp papaya genome [15]. Detailed analysis and annotation of these genomic sequence samples provided initial insights into genome composition, gene number, and other parameters that could help to guide, or in some cases physically anchor, a subsequent complete genome sequencing project.

In a companion paper [16], we examined a random sampling of 30 *F. vesca *genomic sequence segments comprising ~1.0 Mb, or ~0.5% of the *F. vesca *genome. The key resource for that and the present study was a fosmid library from *F. vesca*; constructed from physically sheared and blunt-end cloned genomic DNA fragments from *F. vesca *ssp. *americana *'Pawtuckaway' [17,18]. On the basis of that representative genomic sampling, we concluded that the *F. vesca *genome contains about 30,500 protein-encoding genes, plus > 4700 truncated gene fragments. Over 30 new repeat families were identified, the most common of which were long terminal repeat (LTR) retrotransposons. Total transposable element (TE) content of the *F. vesca *ssp. *americana *genome was estimated to be at least 16%.

In the present study, our aim was to examine a specific set of genomic sequences and gene neighborhoods, targeted for study because they contain genes of likely relevance to horticultural and fruit quality traits, with emphasis on metabolic pathways, flowering-related genes, and disease resistance-related genes. The alcohol dehydrogenase (*ADH*) was targeted on the basis of its use in ongoing phylogenetic studies in *Fragaria *[4,19], and for its historical status as the first protein-encoding gene to be sequenced in strawberry [20]. The granule-bound starch synthase I (*GBSSI*) gene was also of interest relevant to its use in phylogenetic studies of *Fragaria *[5] and the Rosaceae [21].

Our approach necessarily differed from that of the previous study [16], because our focus was on the features of specifically targeted genomic sites rather than on representative genomic sampling. We herein present a detailed description of 20 genomic regions comprising 0.71 Mb of DNA sequence. This description encompasses the identification and annotation of putative genes, pseudogenes, and repetitive elements. It is expected that the results reported herein will be of particular value to plant biologists interested in the studied genes and the traits to which they may relate, and to molecular breeders pursuing the genetic improvement of strawberry and/or other members of the Rosaceae family.

## Results

### Fosmid library

The CTAB isolation procedure produced genomic DNA fragments in the 40 kb size range, evidently a consequence of random, procedure-induced physical shearing. Following end-repair, this genomic DNA was used in a single ligation reaction to construct the fosmid library, which consisted of 33,295 clones arrayed in eighty-seven 384-well plates and spotted in ordered pairs onto multiply replicated sets of two high-density filters.

### Filter hybridizations

Of twenty protein-encoding gene probes used in filter hybridizations, eighteen probes yielded at least one PCR-confirmed target clone. One fosmid clone was selected for each of sixteen probes, while two fosmid clones with distinctly differing restriction fragment patterns were selected for sequencing from each of two probes: gRGA2 and TPS. Thus, a total of 18 protein-encoding gene probes were used to select 20 fosmids (Table 1) to be subcloned and sequenced.

**Table 1:** Features and GenBank accession numbers of sequenced fosmids.

GenBank accession	Fosmid number	Insert length (bp)	Number of contigs	Target gene	Probe name
EU024823	01I13	40,701	2	*HY5*	HY5
EU024826	08G19	38,293	1	*APETALA 3*	AP3
EU024827	10B08	35,178	1	*LEAFY*	LFY
EU024831	13I24	33,654	1	NBS-LRR Resistance-like gene	gRGA2
EU024832	14K06	36,024	1	*ALCOHOL DEHYDROGENASE*	ADH
EU024837	19H07	32,060	3	*SUPPRESSOR OF CONSTANS I*	SOC
EU024838	19M24	32,776	1	NBS-LRR Resistance-like gene	gRGA1
EU024845	32L07	32,968	1	NBS-LRR Resistance-like gene	gRGA2
EU024847	34E24	36,278	1	NBS-LRR Resistance-like gene	gRGA1
EU024852	41O22	32,997	1	*TERPENE SYNTHASE*	TPS
EU024856	48I08	38,603	3	*CHALCONE ISOMERASE*	CHI
EU024859	49M15	42,209	3	LRR Resistance-like gene	gLRR
EU024860	51F10	29,623	1	*PHYTOCHROME A*	PHY
EU024861	52B01	29,916	1	*CONSTANS*	CO
EU024863	52I20	42,439	1	*GRANULE-BOUND STARCH SYNTHASE-I*	GBSSI
EU024864	53J04	32,846	2	*TERPENE SYNTHASE*	TPS
EU024865	53O08	31,107	3	*DIHYDROFLAVONOL 4-REDUCTASE*	DFR
EU024868	73I22	33,392	1	*CHALCONE SYNTHASE*	CHS
EU024870	76C08	44,532	3	*REGULATOR OF ANTHOCYANIN SYNTHESIS*	RAN
EU024871	76K13	32,767	1	*PISTILLATA*	PIST

### Fosmid Insert Sequences

Uninterrupted insert sequences bounded by vector ends were obtained from 13 of 20 fosmid clones. These insert contigs ranged in length from 29,623 bp to 42,439 bp (Table 1). Of the remaining 7 clones, sequence assembly generated two (2 clones) or three (5 clones) ordered contigs (Table 1). For the 7 multi-contig clones, the sum of contig sequence lengths varied from 31,107 bp to 44,532 bp. The total length of insert sequence generated from the 20 selected fosmids was 708,363 bp, or an average of 35,418 bp per insert. These sequences have been deposited into GenBank under the accession numbers listed in Table 1.

### Identification of Genetic Elements

As detailed below, several categories of genetic elements were identified, including protein-encoding genes and pseudogenes, expressed sequences, transposable elements (TEs), and other repetitive elements including simple sequence repeats (SSRs). The results of the bioinformatic analyses are presented in two forms: tabular (Tables 2, 3 and 4 and Additional files 1 and 2) and graphical (Figures 1, 2, 3, 4, 5, 6, 7, 8, 9, 10, 11, 12, 13, 14, 15, 16, 17, 18, 19, 20 and 21). Figure 1 provides a key to the icons, abbreviations, and other conventions employed in the subsequent "Fosmid Figures".

**Table 2:** FGENESH gene predictions and comparisons to homology-based inferences.

FGENESH Model	Predicted genes	Unsupported predictions^1^	Gene mergers^2^	Genes merged^2^	Validated starts^3^	Validated stops^3^	Validated starts + stops
At	159	32	5	10	80	68	148
Mt	163	34	4	8	80	72	152
Mo	115	9	12	27	66	64	130
Nt	208	65	3	7	70	63	133
Le	149	33	8	17	69	65	134
Vv	121	14	16	38	65	62	127

^1^FGENESH predictions were classified as unsupported if no gene was detected at the indicated location via Blast homology searches.

^2^The term "gene merger" refers to instances in which two or more gene sites identified on the basis of Blast homology were merged into a single FGENESH gene prediction. The term "genes merged" refers to the cumulative number of genes involved in such gene mergers.

^3^FGENESH predictions of start and stop codons were considered to be validated if support for their predicted locations was obtained from examination of ORFs in homologous sequences identified by Blast homology searches.

**Table 3:** Instances of micro-colinearity or conserved microsynteny between *F. vesca *and *A. thaliana*.

GenBank	Fosmid	Gene	Putative function	Blastx match	Blastx E value	Arabidopsis locus
EU024823	01I13	4	unknown protein	NP_568247	E = 8.3e-30	At5g11280
EU024823	01I13	5	OCP3 (OVEREXPRESSOR OF CATIONIC PEROXIDASE 3)	NP_196688	E = 4.0e-31	At5g11270
EU024823	01I13	6	HY5 (ELONGATED HYPOCOTYL 5); DNA binding/transcription factor	NP_568246	E = 2.3e-39	At5g11260
EU024823	01I13	7	CESA1 (CELLULOSE SYNTHASE 1); transferase, transferring glycosyl groups	NP_194967	E = 7.0e-160	At4g32410
EU024823	01I13	9	SHS1 (SODIUM HYPERSENSITIVE 1); binding/transporter	NP_194966	E = 1.5e-76	At4g32400
EU024826	08G19	2	floral homeotic protein AP3	NP_191002	E = 7.8e-25	At3g54340
EU024826	08G19	3	putative protein (embryo defective 1967)	NP_566998	E = 1.8e-39	At3g54350
EU024827	10B08	5	ATP-dependent DNA helicase, putative	NP_174109	E = 3.2e-38	At1g27880
EU024827	10B08	6	unknown protein	NP_174106	E = 6.4e-31	At1g27850
EU024831	13I24	3	xanthine/uracil permease family protein	NP_566384	E = 0	At3g10960
EU024831	13I24	4	protein phosphatase-related	NP_566383	E = 9.7e-53	At3g10940
EU024845	32L07	2	SMC2 (STRUCTURAL MAINTENANCE OF CHROMOSOMES 2) (AtSMC2-1) (AtSMC2-2)	NP_201047	E = 5e-83	
				NP_190330	E = 1.0e-80	At5g47460
EU024845	32L07	3	ATNOA1/ATNOS1/NOA1/NOS1 (nitrous oxide synthase 1)	NP_850666	E = 3.0e-34	At3g47450
EU024847	34E24	4	disease resistance protein (CC-NBS-LRR class), putative	NP_201491 NP_201492	E = 5.8e-56 E = 1.0e-54	At5g66900 At5g66910
EU024847	34E24	6	disease resistance protein (CC-NBS-LRR class), putative	NP_201491 NP_201492	E = 6.5e-63 E = 1.2e-59	At5g66900 At5g66910
EU024847	34E24	7	disease resistance protein (CC-NBS-LRR class), putative	NP_201491 NP_201492	E = 3.9e-90 E = 1.9e-89	At5g66900 At5g66910
EU024852	41O22	2	terpene synthase/cyclase family protein	NP_176361	E = 2.1e-55	At1g61680
EU024852	41O22	4	terpene synthase/cyclase family protein	NP_176361	E = 2.4e-56	At1g61680
EU024852	41O22	5	zinc ion binding	NP_176362	E = 8.0e-164	At1g61690
EU024856	48I08	1	protein kinase, putative	NP_190214	E = 2.5e-153	At3g46290
EU024856	48I08	2	protein kinase-related	NP_190213	E = 1.2e-25	At3g46280
EU024856	48I08	3	protein kinase-related	NP_190213	E = 6.6e-9	At3g46280
EU024860	51F10	1	EMB1138 (EMBRYO DEFECTIVE 1138); ATP binding/ATP-dependent helicase	NP_001031943	E = 7.0e-82	At5g26742
EU024860	51F10	3	unknown protein	NP_568485	E = 1.0e-8	At5g26731
EU024860	51F10	2	unknown protein	NP_187245	E = 8.2e-27	At3g05940
EU024860	51F10	4	unknown protein	NP_001118586	E = 1.3e-8	At3g05936
EU024863	52I20	1	oxidoreductase	NP_568767	E = 2.3e-25	At5g51880
EU024863	52I20	4	unknown protein	NP_568766	E = 1.7e-9	At5g51840
EU024863	52I20	2	AGL42 (AGAMOUS LIKE 42); transcription factor	NP_568952	E = 1.3e-22	At5g62165
EU024863	52I20	3	unknown protein	NP_201023	E = 6.9e-19	At5g62170
EU024863	52I20	6a	DNA binding/binding/protein binding/zinc ion binding	NP_567188	E = 1.2e-44	At4g00790
EU024863	52I20	6b	unknown protein	NP_567189	E = 0	At4g00800
EU024864	53J04	3	unknown protein	NP_564783	E = 8.5e-41	At1g61667
EU024864	53J04	6	terpene synthase/cyclase family protein	NP_176361	E = 1.2e-123	At1g61680
EU024871	76K13	4	PTAC6 (PLASTID TRANSCRIPTIONALLY ACTIVE6)	At: NP_564144	E = 2.6e-54	At1g21600
EU024871	76K13	6	wound-responsive family protein	At: NP_173580	E = 3.9e-14	At1g21610

**Table 4:** Summary of SSR data.

SSR motif	Number of SSR loci	SSRs per Mb
**Di-nucleotide**	123	174
AG	58	82
AT	48	68
AC	17	24
**Tri-nucleotide**	35	49
AGG	5	7
ACC	2	3
ATT	4	6
GAA	14	20
GTT	4	6
ACT	4	6
AGT	2	3
**Tetra-nucleotide**	0	0
**Penta-nucleotide**	0	0

**TOTAL**	158	223

**Figure 1:**
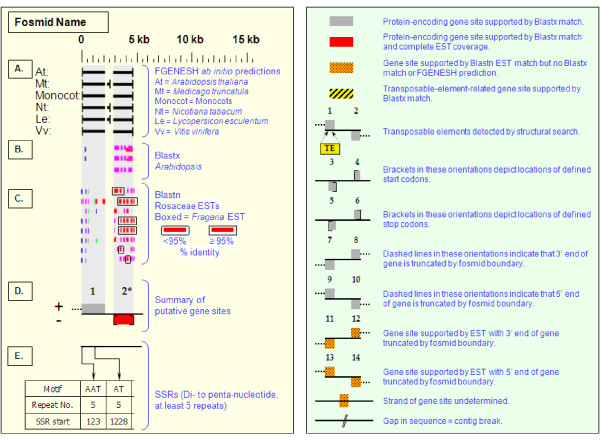
**Fosmid Key**. The gene space diagrams that accompany this text share a common format for presentation of figures. Analysis of fosmids includes five horizontal panels: A through E (as depicted on the left side of the figure). Panel A graphically depicts the *ab initio *gene location and orientation predictions of the six FGENESH training models. Panel B depicts the outputs of Blastx searches of the GenBank non-redundant protein database, delimited to Arabidopsis. Panel C depicts the outputs of Blastn comparisons against Rosaceae ESTs. Panel D depicts the inferred locations and orientations of the putative gene sites inferred on the basis of Blastx homology-based analysis, and also depicts the locations of transposable element sites detected by structural analysis. Panel E provides a description of SSR content as detected by the SSRIT analysis. The information to the right provides a range of descriptive information that is found throughout Panels A-E. On the right side of the figure, the various icons used in the Fosmid Figures are defined.

**Figure 2:**
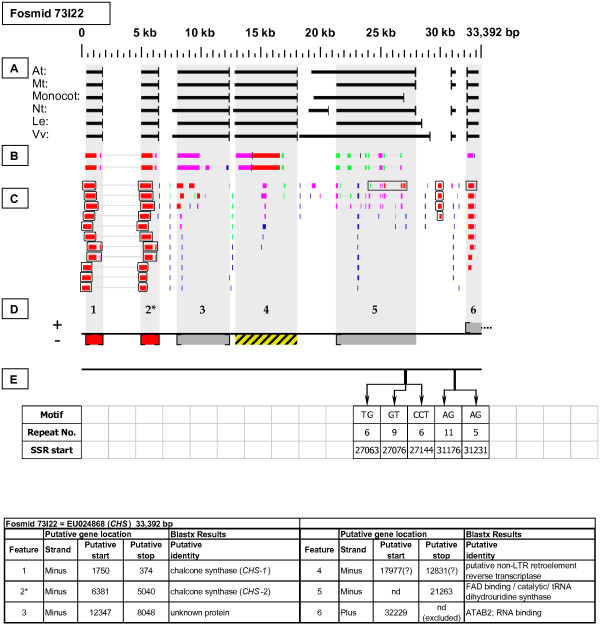
***Chalcone synthase *gene neighborhood**. On fosmid 73I22, the targeted *CHS *gene is tandemly duplicated (as gene sites 1 and 2). Both *CHS *genes have complete, top-tier F-EST coverage, and were modeled. Gene site 4 encodes a putative transposable-element-related protein. Gene site 6 is artifactually truncated at its 3' end by the fosmid insert boundary.

**Figure 3:**
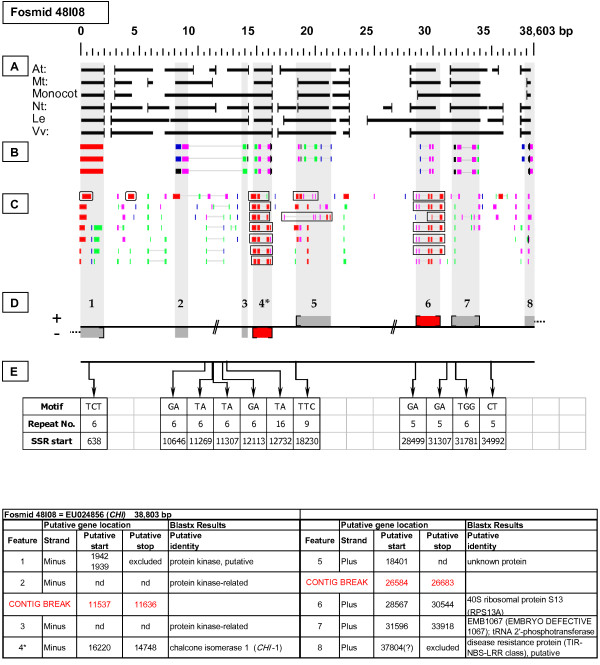
***Chalcone isomerase *gene neighborhood**. On fosmid 48I08, the targeted *CHI *gene resides at gene site 4. It has complete, top-tier F-EST coverage. The 40S ribosomal protein (RPS13A) gene at site 6 has complete, top-tier F-EST coverage. Both of these genes were modeled. Three protein kinase genes and/or gene fragments reside sequentially adjacent to the *CHI *gene. Gene sites 1 and 8 are each truncated by the fosmid insert boundaries. The predicted gene product of site 8 is a TIR-NBS-LRR type disease resistance-like gene.

**Figure 4:**
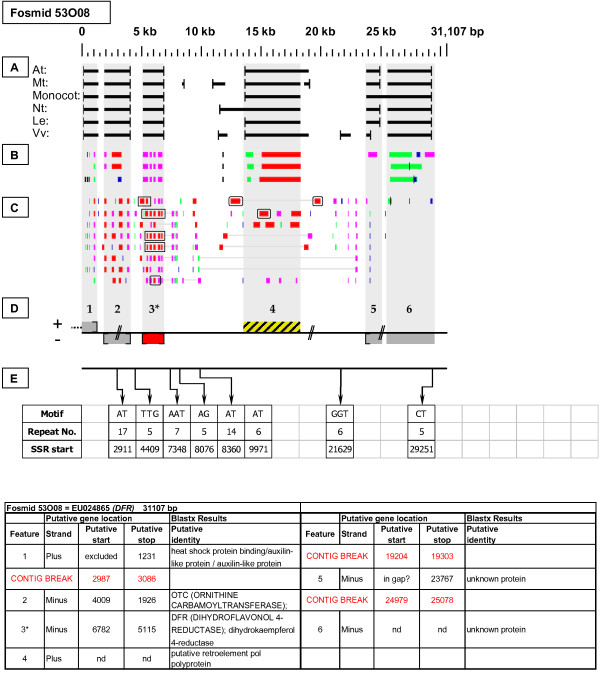
***Dihydroflavonol 4-Reductase *gene neighborhood**. On fosmid 53O08, the targeted *DFR *gene resides at gene site 3. It has complete, top-tier F-EST coverage, and was modeled. Gene site 4 encodes a putative transposable-element-related protein. The sequence at gene sites 2 and 5 are interrupted by contig breaks. Gene site 1 is truncated by the fosmid insert boundary.

**Figure 5:**
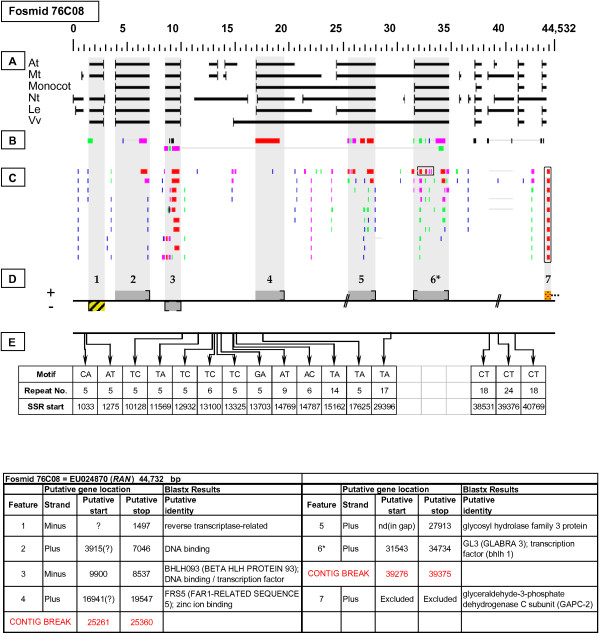
***Regulator of Anthocyanin Biosynthesis *gene neighborhood**. On fosmid 76C08, the targeted *RAN *gene resides at gene site 6 [*GL3 *(*GLABRA 3*); transcription factor (bHLH 1)]. It has partial top-tier F-EST coverage. Gene site 1 encodes a putative transposable-element-related protein. The sequence at gene site 7 is interrupted by the fosmid insert boundary: here, only the 5' UTR of the indicated gene is present within the insert boundary.

**Figure 6:**
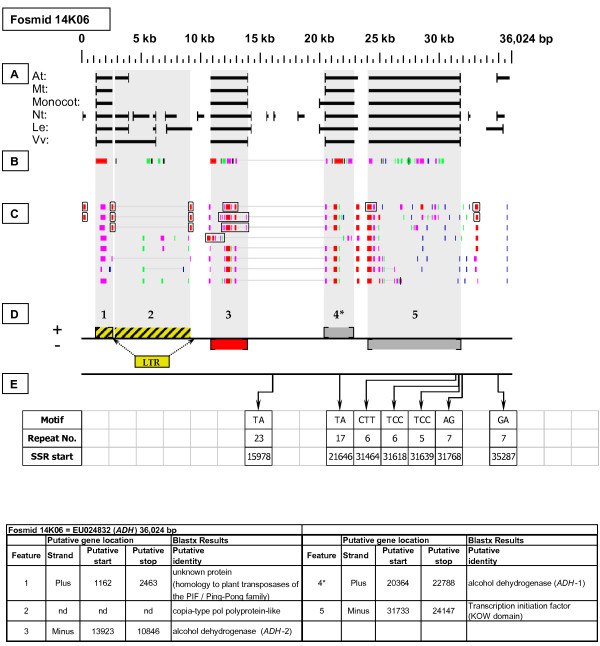
***Alcohol Dehydrogenase *gene neighborhood**. The targeted gene, *ADH-*1, is located on fosmid 14K06 at gene site 4. It has no top-tier F-EST coverage. A second *ADH *gene, *ADH*-2 resides at gene site 3. It has complete top-tier F-EST coverage, and was modeled. Gene sites 1 and 2 encode transposable-element-related proteins, and site 2 was identified as an LTR-retrotransposon.

**Figure 7:**
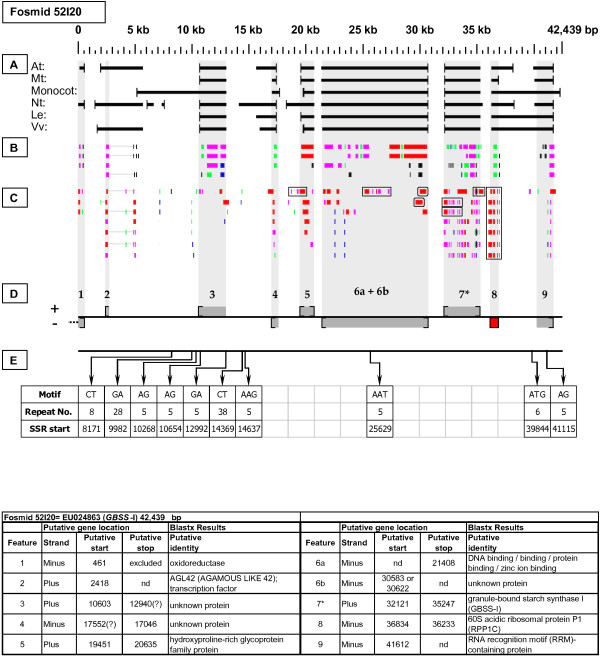
***Granule-Bound Starch Synthase-I *gene neighborhood**. On fosmid 52I20, the targeted *GBSSI *gene resides at gene site 7. It has partial, top-tier F-EST coverage. Gene sites 6a and 6b ostensibly encode distinctly different proteins; yet the stop codon of 6b and the start codon of 6a could not be determined, and a bridging, top-tier F-EST [GenBank: DY672167] seems to tie 6a and 6b together transcriptionally (see boxed EST in 6a-6b region, Fig. 7). Interestingly, the Arabidopsis homologs of genes 6a and 6b are colinear with them (Table 3). The 60S ribosomal protein (RPP1C) gene at site 8 has complete, top-tier F-EST coverage, and was modeled.

**Figure 8:**
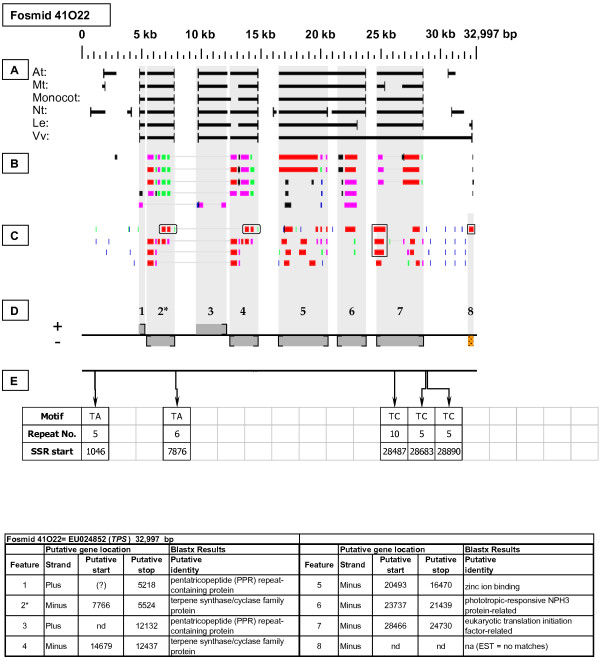
***Terpene Synthase *gene neighborhood (one of two)**. On fosmid 41O22, two copies of the targeted *TPS *gene reside at gene sites 2 and 4, of which the gene site 2 sequence best matches that of the hybridization probe. Both *TPS *genes lack top-tier F-EST coverage. Each *TPS *gene has as its immediate neighbor a pentatricopeptide (*PPR*) gene (sites 1 and 3), constituting a tandem duplication of the *TPS*-*PPR *gene pair.

**Figure 9:**
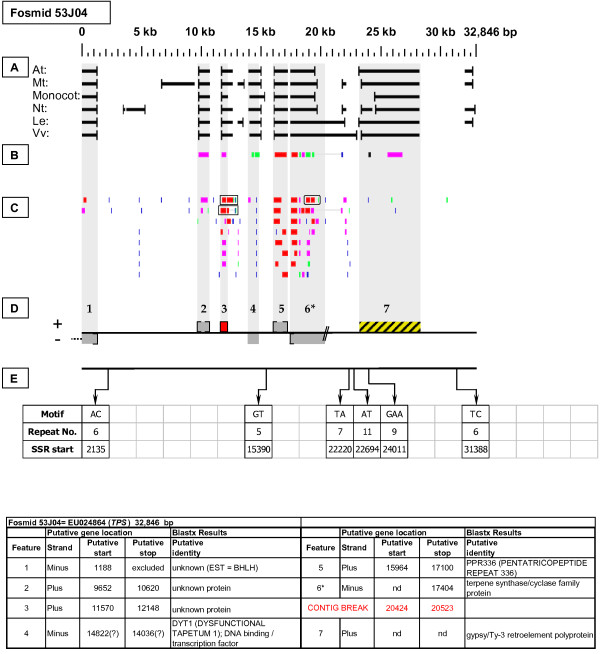
***Terpene Synthase *gene neighborhood (one of two)**. On fosmid 53J04, a single copy of the targeted *TPS *gene resides at gene site 6. The 5' end of this gene's sequence is artifactually truncated by a contig break, precluding establishment of the start codon location. The *TPS *gene has as its immediate neighbor a pentatricopeptide (*PPR*) gene (site 5). The gene of unknown function at site 3 has complete top-tier F-EST coverage, and was modeled. Gene site 7 encodes a putative transposable-element-related protein.

**Figure 10:**
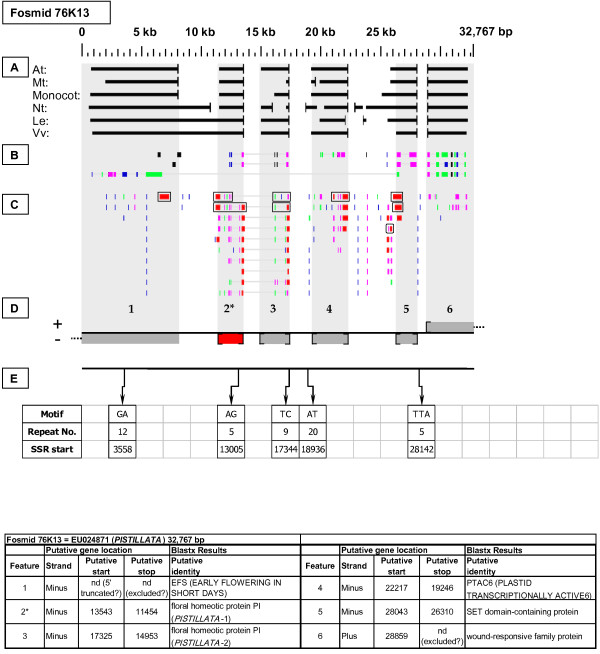
***PISTILLATA *gene neighborhood**. On fosmid 76K13, the targeted *PISTILLATA *gene is tandemly duplicated (as gene sites 2 and 3). The *PISTILLATA *copy at gene site 2 best matches the sequence of the hybridization probe. It has complete, top-tier F-EST coverage, and was modeled. The copy at gene site 3 lacks complete top-tier F-EST coverage.

**Figure 11:**
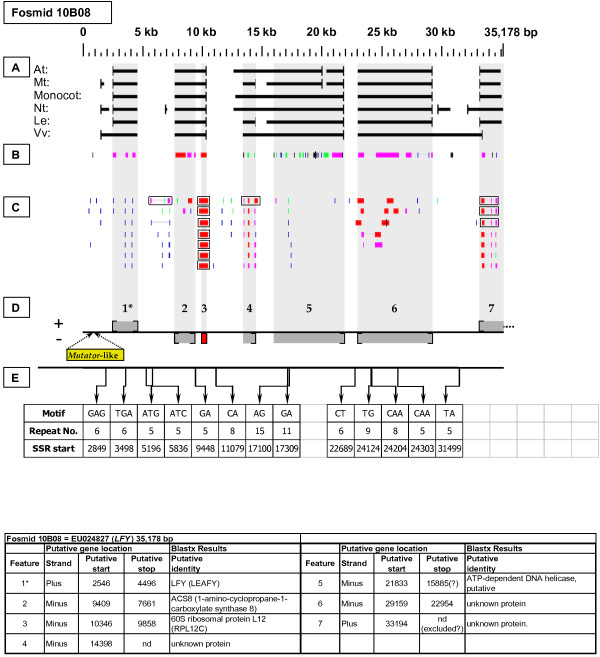
***LEAFY *gene neighborhood**. The targeted *LFY *gene is located on fosmid 10B08 at gene site 1. It has no top-tier F-EST coverage. A *Mutator*-like repetitive element is located just upstream of the *LFY *gene. The 60S ribosomal protein (RPL12C) gene at site 3 has complete, top-tier F-EST coverage, and was modeled. Note that all FGENESH training models merged two distinct gene sites, 2 and 3, into a single gene prediction.

**Figure 12:**
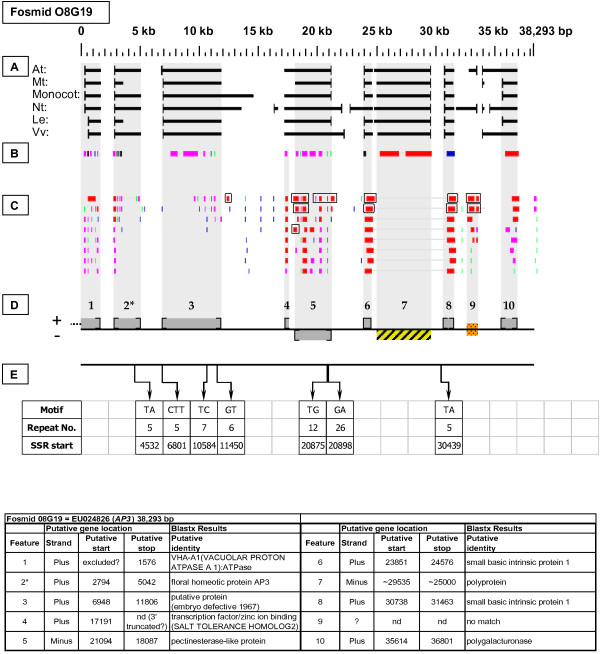
***APETALA3 *gene neighborhood**. The targeted *AP3 *gene is located on fosmid 08G19 at gene site 2. It has no top-tier F-EST coverage. Gene site 7 encodes a putative transposable-element-related protein. Gene site 9 had no good quality Blastx hits, but had two top-tier F-EST matches [GenBank: DY667692 and DY674185], suggesting that this putative gene site is transcribed.

**Figure 13:**
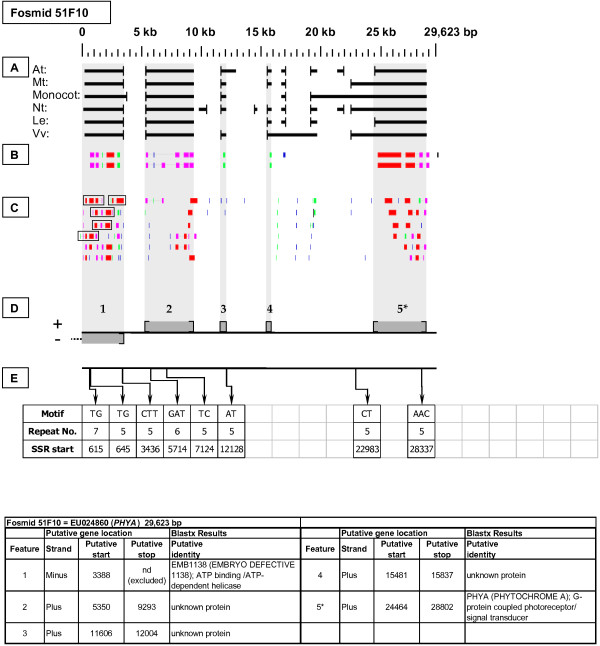
***Phytochrome A *gene neighborhood**. The targeted gene, *PHYA*, resides at gene site 5 in fosmid 51F10. It had no top-tier F-EST coverage.

**Figure 14:**
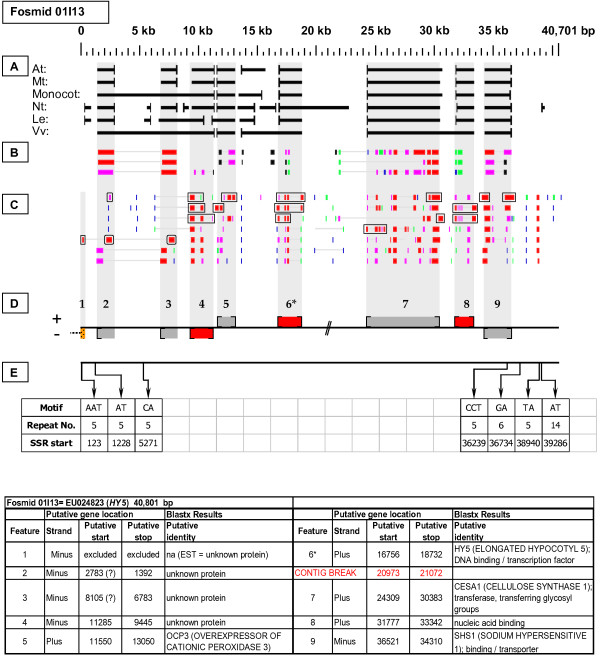
***Elongated Hypocotyl 5 gene *neighborhood**. The targeted gene, *HY5*, resides at site 6 in fosmid 01I13. It has complete, top-tier F-EST coverage, as do gene sites 4 (unknown protein) and 8 (*CELLULOSE SYNTHASE I*). These three genes were modeled.

**Figure 15:**
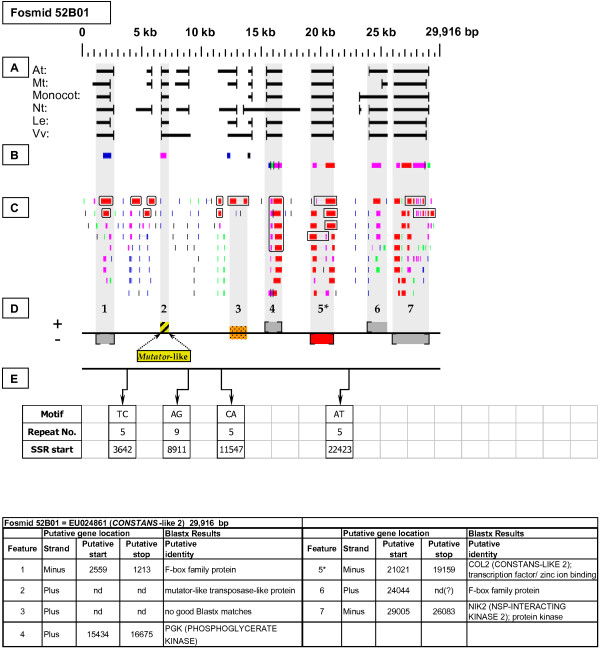
***CONSTANS-LIKE 2 *gene neighborhood**. The targeted *CONSTANS-*like gene resides at gene site 5 in fosmid 52B01. It has complete, top-tier F-EST coverage, and was modeled. Gene site 2 is a *Mutator*-like repetitive element and encodes a putative transposable-element-related protein. Gene site 3 had no good quality Blastx hit, but had one top-tier F-EST match [GenBank: DY670320], suggesting that this putative gene site is transcribed.

**Figure 16:**
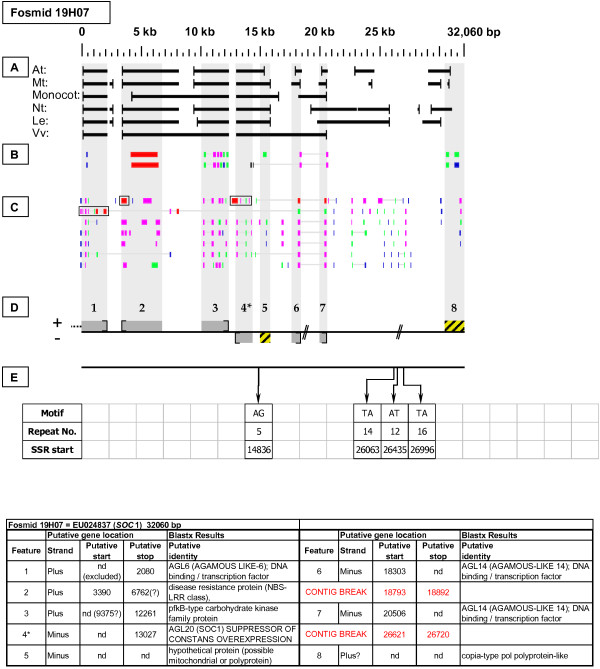
***Suppressor of Constans Overexpression *gene neighborhood**. On fosmid 19H07, the targeted *SOC*1 gene is located at site 4. It has one top-tier F-EST match that does not provide complete EST coverage. Gene sites 5 and 8 encode putative transposable-element-like proteins.

**Figure 17:**
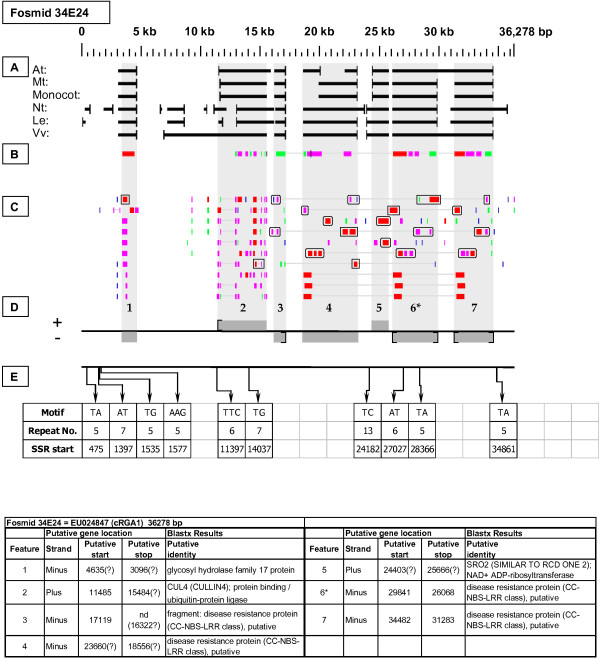
**cRGA1 Resistance-like gene neighborhood**. Fosmid 34E24 contains a cluster of NBS-LRR disease resistance-like genes, one a fragment (site 3) and three evidently full length (sites 4,6, and 7), of which the site 6 gene was the best match to the hybridization probe. The genes at sites 4 and 6 have, respectively, one and two top-tier F-EST matches, providing evidence that these sites are transcribed.

**Figure 18:**
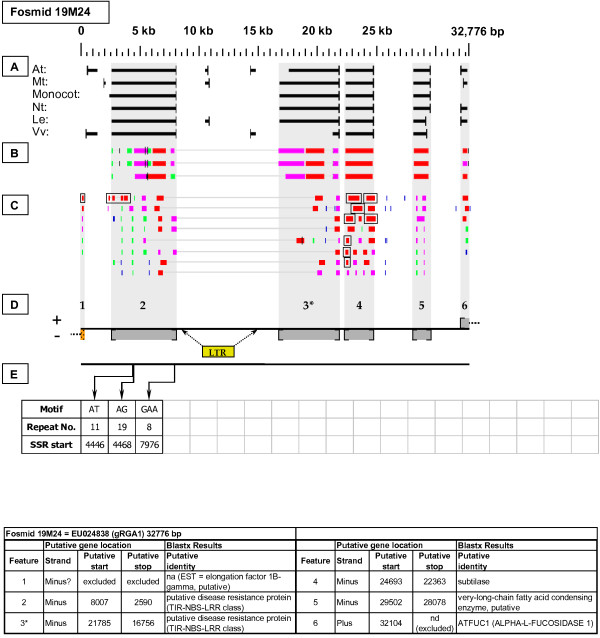
**gRGA1 Resistance-like gene neighborhood**. Fosmid 19M24 contains a pair of apparently full length NBS-LRR disease resistance-like genes (sites 2 and 3), of which the site 3 gene was the best match to the hybridization probe. Site 2 has one top-tier F-EST match that does not provide complete EST coverage, but suggests that this gene site is transcribed. Site 3 had no top-tier F-EST coverage. An LTR retrotransposon was identified in the region between gene sites 2 and 3. Gene site 4 has complete top-tier F-EST coverage, suggesting that this gene site is transcribed.

**Figure 19:**
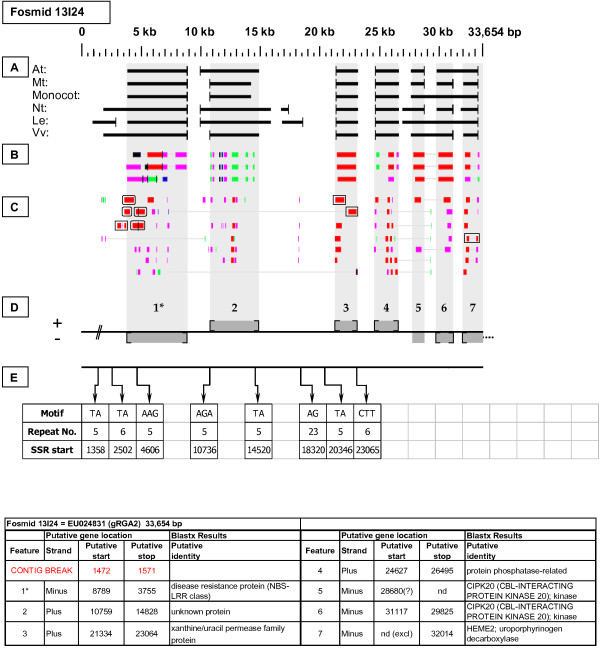
**gRGA2 Resistance-like gene neighborhood (1)**. In fosmid 13I24, the targeted NBS-LRR resistance-like gene is located at gene site 1.

**Figure 20:**
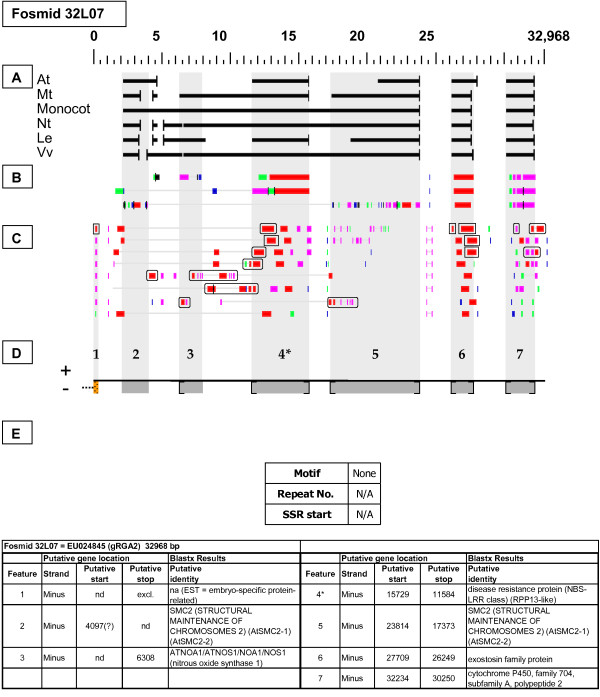
**gRGA2 Resistance-like gene neighborhood (2)**. In fosmid 32L07, the targeted NBS-LRR resistance-like gene (RPP13-like) is located at gene site 4. Site 4 has one top-tier F-EST match that does not provide complete EST coverage, but suggests that this gene site is transcribed. This fosmid was unique in containing no SSR loci.

**Figure 21:**
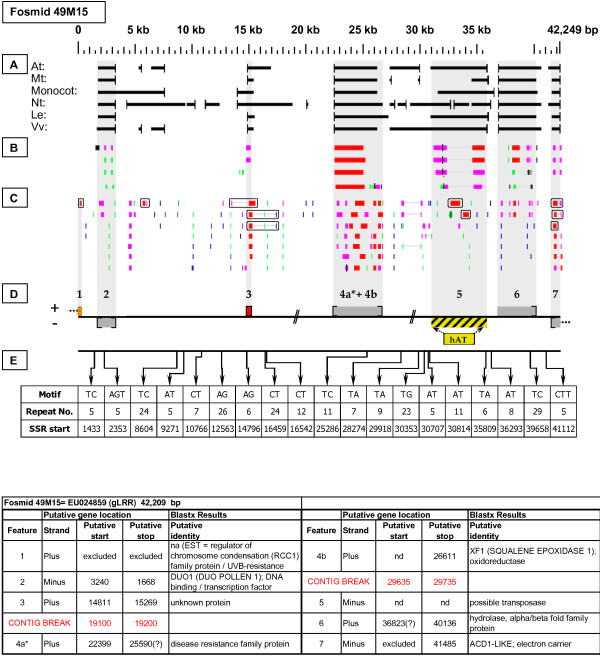
**gLRR Resistance-like gene neighborhood**. On fosmid 49M15, the evident probe target was a disease resistance protein family gene at site 4a. Site 4b has Blastx homology to a squalene epoxidase gene. Both genes are on the plus strand. Neither the stop codon of gene 4a nor the start codon of gene 4b could be confidently determined. Moreover, the two respective coding regions are bridged by a matching 1447 bp *Malus *cDNA sequence [GenBank: EG631376], suggesting that region 4a + 4b may be transcribed as a single unit. Note that regions 4a-4b are merged into one gene prediction by all six FGENESH models. Gene site 5 encodes a putative transposable-element-related protein, and is the site of an hAT repetitive element. Gene site 3 (unknown protein) has complete, top-tier F-EST coverage, and was modeled.

Each Fosmid Figure consists of five parts (see Figure 2). Part A depicts the *ab initio *predictions of the six FGENESH training models. Part B depicts the output of Blastx homology searches of the NCBI protein database, delimited to Arabidopsis or Viridiplantae. Part C depicts the output of Blastn homology searches of the NCBI EST-Others database, delimited to the Rosaceae family. *Fragaria *EST matches are boxed. Part D provides a graphic representation of the annotated features of the respective fosmid insert, wherein each icon-coded gene site is coupled to its supporting evidence and FGENESH predictions (if any) by a vertical, gray stripe. Part E depicts the locations, sequence motifs, and repeat numbers of SSR loci. Finally, each Fosmid Figure is accompanied by an abbreviated spreadsheet that provides information about the annotated gene sites, including strand assignments, putative start and stop codon sites, and putative gene product identity. A more detailed listing of the homology-based evidence supporting each gene site identification is provided in Additional file 1: Fosmid Gene Content Annotation.

### *Ab initio *predictions (Fosmid Figures: Part A)

The number of predicted protein-encoding genes varied considerably among the six FGENESH models (Table 2), ranging from a low of 115 (Mt model) to a high of 208 (Nt model), with an overall mean of 152.5. As detailed below, the subsequent generation of a set of homology-based inferences provided a basis for comparison with the outputs of the six *ab initio *models.

### Blastx homology (Fosmid Figures: Part B)

Blastx searches of the NCBI Arabidopsis and Viridiplantae protein databases, employing the fosmid inserts as query sequences, were used to identify protein-encoding gene sites, including intact genes, pseudogenes, and TE-related proteins. Using a conservative cutoff of e = 10^-10 ^[16], a total of 123 gene sites were identified, while a less conservative cutoff of e = 10^-5 ^yielded eight additional gene sites. With reference to their best Blastx matches, 11 gene sites encoded TE-related proteins (reverse transcriptase, transposase, polyprotein, etc.), 100 encoded proteins with tentative or definitive functional identities, and 20 encoded "unknown proteins". The inferred strand assignments of each of these 131 genes sites are provided in the Fosmid Figures and in Additional file 1: Fosmid Gene Content Annotation, Column C.

### Blastn homology to *Fragaria *and Rosaceae ESTs (Fosmid Figures: Part C)

In total, Blastn searches of the NCBI Rosaceae EST database (conducted prior to June 1, 2009) recovered 128 "top-tier" (≥ 95% sequence identity) EST matches, of which 126 were from *Fragaria *(F-ESTs) [Additional file 1: Fosmid Gene Content Annotation, Columns K, L, M, N, O and P] and two were from *Rosa*. Top-tier F-EST coverage varied considerably among the 131 Blastx-inferred gene sites. Fifty-five gene sites had one or more top-tier F-EST matches, while 76 had none. Fifteen genes sites had complete EST coverage [Additional file 1: Fosmid Gene Content Annotation - coded in red], providing a basis for establishing gene models, as addressed below. Numerous "second-tier" (85-94% identity range) ESTs from *Fragaria *and other rosaceous species (including *Malus*, *Prunus*, *Rosa, Rubus*, and *Pyrus*) occurred at many of the identified gene sites [Additional file 1: Fosmid Gene Content Annotation, Columns Q, R and S], and elsewhere within the fosmid sequences. The second tier F-ESTs were denoted by rounded boxes in the Fosmid Figures.

Several top-tier F-EST matches occurred at genomic sites that lacked Blastx matches and FGENESH predictions. Six such gene sites occurred at fosmid insert boundaries, where the match comprised a 5' or 3' UTR sequence but little or no coding sequence. In the absence of recognizable coding sequence within the fosmid boundaries, these putative gene sites were invisible to FGENESH and Blastx. Additionally, two F-EST sites that lacked Blastx matches and FGENESH predictions occurred internally in the inserts. Gene site 9 in fosmid 08G19 (Figure 12) was defined by F-ESTs [GenBank: DY667692 and DY674185], and gene site 3 in fosmid 52B01 (Figure 15) was also defined by an F-EST [GenBank: DY670302]. These F-ESTs lacked obvious ORFS, and Blastx searches using the ESTs themselves as queries obtained no quality matches. Thus, the F-ESTs in question may be non-coding, or may encode previously undescribed proteins.

### Comparison of *ab initio *predictions and homology-based inferences

Homology-based inferences of gene site numbers and locations and of start and stop codon positions provided a basis for evaluating the differing outputs of the six FGENESH *ab initio *models. As previously noted, the number of predicted genes varied considerably among *ab initio *models (Table 2). As compared with the number of 131 Blastx inferred gene sites, the Mo (115) and Vv (121) models under-predicted, while the remaining models over-predicted the number of protein-encoding genetic elements.

Under-prediction was primarily due to "gene merging": the prediction of one large gene over a region wherein two or more separate genes were detected by homology search (e.g., Figure 11, gene sites 2 and 3). The Vv model generated a high of 16 such "gene mergers", involving a total of 38 Blastx-inferred genes, while the Nt model generated only 3 gene mergers, involving 7 Blastx-inferred genes (Table 2). Over-prediction was primarily due to absence of homology-based evidence of gene location at some sites of *ab initio *prediction. The number of non-validated *ab initio *predictions ranged from a high of 65 (Nt) to a low of 9 (Mo) (Table 2).

For each of the Blastx-identified genes, start and stop codon positions were inferred on the basis of homology searches supplemented by manual examination of reading frames as displayed by SeqBuilder (DNAStar), utilizing comparisons to F-ESTs wherever possible. This homology-based analysis established the putative locations of 93 start and 92 stop codons.

The discrepancy between the homology based inference of 131 gene sites and the substantially lesser numbers of inferred start and stop codon locations was attributable to several factors. Nineteen of the 131 gene sites were artifactually truncated at their 5' or 3' ends, either by a fosmid end or by a contig gap. Because no contig gaps interrupted genes of specific interest, effort was not invested in closing these gaps, with the expectation that they soon will be closed by the anticipated whole genome sequencing of *Fragaria vesca*. Another obstacle was "biological truncation" [16], in which a terminal segment of a coding region is missing due to deletion or rearrangement. As many as 20 of the identified gene sites appeared to be truncated pseudogenes, as evidenced by substantial shortening of the predicted protein as compared with its putative Arabidopsis homolog. Of the 11 TE-related gene sites, only one start codon and two stop codons could be identified. Lack of EST support also precluded establishment of start and/or stop codon positions in many genes.

When the homology-inferred 93 start and 92 stop codon locations were compared to those predicted for the respective genes by the FGENESH models (Table 2), the highest number of start and stop codon agreements were, respectively, 80 (At and Mt) and 72 (Mt). The lowest numbers, respectively, were 65 (Vv) and 62 (Vv). Among the six FGENESH models, the closest overall agreement with our homology-based inferences of start and stop codon positions was provided by the Mt model, with the At model a close second best (Table 2).

### Gene Models

Using the GeneSeqer program, intron locations were determined in each of the 15 genes for which complete coding sequence (cds) EST coverage was available. This information, in combination with previously detailed determinations of start and stop codon locations, provided a basis for defining gene models [Additional file 2: Gene Models]. Among the 15 modeled genes, intron number ranged from zero to nine per gene, with a mean of 2.87. The intron size range, from 84 bp to 933 bp, approximated a Poisson distribution, with 23 of 43 introns falling below 150 bp in length and 30 falling below the mean intron size of 289 bp [Additional file 2: Gene Models].

### Targeted genes

A central objective of this project was to obtain the complete genomic sequences of a suite of targeted genes that were of potentially broad interest to the *Fragaria*/Rosaceae research community and industry. Each fosmid clone contained a gene site that clearly corresponded, with ≥ 98% nucleotide sequence identity, to its respective hybridization probe. Each of the targeted genes was contained entirely within the respective fosmid boundaries, providing knowledge of promoter and other flanking regulatory sequences. Noteworthy features of these targeted genes are described below, with the expectation that they will be the subjects of intensive follow-up studies.

### Anthocyanin-pathway-related genes

#### Chalcone Synthase (CHS)

Two adjacent copies of the *CHS *gene are present in head-to-tail orientation on minus strand of fosmid 73I22 (Figure 2). For purposes of discussion only, we provisionally designate the downstream and upstream gene copies as *CHS*-1 and *CHS*-2, respectively. Six F-ESTs were identified that provide top-tier matches to one or both of these gene copies. Three F-ESTs best match *CHS*-1, and three best match *CHS*-2, uniquely providing full F-EST coverage to each gene copy. The resulting gene models, each displaying the presence of one intron, are provided in Additional file 2: Gene Models. A comparison of *CHS*-1 and *CHS*-2 coding sequences showed them to have 95% nucleotide sequence identity and 98% predicted amino acid sequence identity. In contrast, the nucleotide sequences of the single intron of each gene shared only 55% identity.

#### Chalcone Isomerase (CHI)

Fosmid 48I08 (Figure 3) contains a single *CHI *gene (site 4). Two top-tier F-ESTs provide complete coding sequence coverage, with the resulting gene model displaying the presence of three introns [Additional file 2: Gene Models].

#### Dihydroflavonol 4-Reductase (DFR)

Fosmid 53O08 (Figure 4) contains a single *DFR *gene. Four top-tier F-ESTs provide complete cds coverage, with the resulting gene model displaying the presence of five introns [Additional file 2: Gene Models].

#### Regulator of Anthocyanin Synthesis (RAN)

Fosmid 76C08 (Figure 5) was selected by a probe targeted to a *Del*-like regulatory gene designated as *RAN *in strawberry [22]. The probe sequence has 98% nucleotide identity to a region of gene site 6 on the same fosmid. In taxon-delimited Blastx searches, this gene site had the following best protein matches: GL3 (GLABRA 3) and transcription factor (bHLH 1) (Arabidopsis); and myc-like anthocyanin regulatory protein (Viridiplantae). One top-tier F-EST match provides partial EST coverage for this gene.

### Other metabolism-related genes

#### Alcohol Dehydrogenase (ADH)

Two adjacent copies of the targeted *ADH *gene are present in head-to-head orientation on fosmid 14K06 (Figure 6). The gene copy on the plus strand (site 4) corresponds to the originally sequenced *ADH *gene from *F*. ×*ananassa *[20], in recognition of which we designate it *ADH*-1. This gene copy had no F-EST support. The gene copy on the minus strand (site 3), which we designate *ADH*-2, had four matching F-ESTs, providing full coding sequence coverage. The resulting gene model is provided in Additional file 2: Gene Models. There is 85% identity between the inferred amino acid sequence of *ADH*-2, and the previously defined amino acid sequence of *ADH*-1 [20], while the coding sequences of these two genes have 77% nucleotide identity. Both *ADH*-1 and *ADH*-2 have nine introns, and between the two genes the corresponding intron sequences are so divergent as to preclude meaningful alignment.

#### Granule-Bound Starch Synthase-I (GBSSI)

Fosmid 52I20 (Figure 7) contains a single *GBSSI *gene (site 7). Of the two *GBSSI *genes known in *Fragaria *(5), this is *GBSSI*-1. Three top-tier F-ESTs provide EST coverage of this gene's 5' and 3' ends.

#### Terpene Synthase (TPS)

Two fosmids with differing restriction digestion patterns were selected by their hybridization to the *TPS *probe. Fosmid 41O22 (Figure 8) contains two *TPS *genes (sites 2 and 4), while fosmid 53J04 (Figure 9) contains one *TPS *gene (site 6). None of the three identified *TPS *genes have top-tier F-EST matches. A single *Fragaria *EST Blastn match [GenBank: CO817558], has only 94% nucleotide sequence identity to each of the three identified *TPS *genes, suggesting that this EST may be the transcript of an additional *TPS *gene in the *Fragaria *genome. Intriguingly, each *TPS *gene has as its immediate downstream neighbor a pentatricopeptide (*PPR*) gene, in tail-to-tail orientation. In fosmid 40I22 the 5' ends of both *PPR *genes appear to be truncated, while the *PPR *gene in fosmid 53J04 appears to have a full length coding sequence.

### Flowering-related genes

#### Pistillata (PI)

Two adjacent copies of the *PISTILLATA *gene were present in head-to-tail orientation on the minus strand of fosmid 76K13 (Figure 10). The gene copy at site 2, which we designate as *PISTILLATA*-1, had two matching top-tier F-ESTs, providing full coding sequence coverage. The *PISTILLATA*-1 gene model, which contains six introns, is presented in Additional file 2: Gene Models. The gene copy at site 3, designated *PISTILLATA*-2, had no matching F-ESTs. A partial *Fragaria *homolog of the Arabidopsis *EFS *(*EARLY FLOWERING IN SHORT DAYS*) gene resides at gene site 1. Thus, fosmid 76K13 captures members of a multi-gene cluster that may play a complex role in the regulation of flowering in *Fragaria*. Notably, in Arabidopsis the *PISTILLATA *gene is not tandemly duplicated, and it resides at a locus (At5g20240) unlinked to that of the *EFS *gene (At1g77300).

#### Leafy (LFY)

Fosmid 10B08 (Figure 11) contains a single copy of the *LEAFY *gene (site 1). This gene had no top-tier F-EST matches, or any high percentage EST matches in any Rosaceae species.

#### Apetala3 (AP3)

Fosmid 08G19 (Figure 12) contains a single copy of the *AP3 *gene (site 2). This gene had no top-tier F-EST matches.

#### Phytochrome A (PHYA)

Fosmid 51F10 (Figure 13) contains a single copy of the *PHYA *gene (site 5). This gene had no top-tier F-EST matches.

#### Elongated Hypocotyl 5 (HY5)

Fosmid 01I13 (Figure 14) contains a single copy of the *HY5 *gene (site 6). Three top-tier F-ESTs provide complete cds coverage. The *HY5 *gene model, which contains two introns, is presented in Additional file 2: Gene Models.

#### Constans (CO)

Fosmid 52B01 (Figure 15) contains a single copy of a targeted gene (site 5) that has the COL-2 (CONSTANS-LIKE 2) protein as its best Arabidopsis Blastx match. Four top-tier F-ESTs provide complete coding sequence coverage. The respective gene model, which contains one intron, is presented in Additional file 2: Gene Models.

#### Suppressor of Constans Overexpression (SOC)

In fosmid 19H07 (Figure 16), the probe hybridization was to gene site 4, which has the Arabidopsis AGL20 (SOC1) SUPPRESSOR OF CONSTANS OVEREXPRESSION 1 protein as its best Arabidopsis Blastx match. This gene has one top-tier F-EST match that provides partial coding sequence coverage, but not of the 5' end, thus leaving the start codon site undetermined. Also on fosmid 19H07, gene sites 6 and 7 both have best Blastx homology to the Arabidopsis *AGL*6 (*AGAMOUS LIKE-*6) gene, but appear to be truncated at their 3' ends and have no top-tier F-EST matches. Yet interestingly, a *Fragaria vesca *SOC 1 protein sequence [GenBank: ACR24128 see http://www.ncbi.nlm.nih.gov/protein] identified by Blastx search, has very high homology at its C terminal end to gene site 4, and at its N terminal end to gene sites 6 and 7. Thus, gene site(s) 6 and/or and 7 may actually constitute the 5' end(s) of the *F. vesca SOC *gene - a hypothesis that will attract further investigation.

### NBS-LRR disease resistance-like genes

#### cRGA1

Fosmid 34E24 (Figure 17) contains a cluster of NBS-LRR disease resistance-like genes. Three of these are apparently full length NBS-LRR resistance-like genes (sites 4, 6, and 7), each of which has strong Blastx homology to the protein products of both members of a tandemly duplicated pair of Arabidopsis loci: At5g66900 and At5g66910. Additionally, gene site 3 is an apparently truncated resistance-like gene. Two F-ESTs provide top-tier matches to the internal region of gene site 4, while two other F-ESTs (including the probe source [GenBank: DV439384]) provide top-tier matches to the 5' and 3' ends of gene site 6, providing evidence that these two genes are transcribed.

#### gRGA1

Fosmid 19M24 (Figure 18) contains a pair of NBS-LRR disease resistance-like genes (sites 2 and 3). Both have among their top Blastx matches an Arabidopsis NBS-LRR resistance-like protein encoded by locus At4g12010. One F-EST provides a top-tier match to the 3' end of gene site 2.

#### gRGA2

Two fosmids with differing restriction digestion patterns were selected by their hybridization to the genomic *gRGA2 *probe. In fosmid 13I24 (Figure 19), the probe target is gene site 1. This gene has one marginally top-tier F-EST match (95.08% identity) that provides coverage mostly of the 3' UTR. In fosmid 32L07 (Figure 20), the probe target is gene site 4, which has one top-tier F-EST match. The indicated gene sites on both fosmids show sequence similarity to NBS-LRR resistance-like proteins encoded by both members of tandemly duplicated Arabidopsis loci At3g14460 and At3g14470.

### LRR disease resistance-like gene

#### gLRR

Fosmid 49M15 (Figure 21) was selected by its hybridization to the probe gLRR at gene site 4a. This gene site had as its best Blastx match a disease resistance family protein encoded by Arabidopsis locus At2g34930, and had no top-tier F-EST matches. The 3' structure of this gene is ambiguous, with evidence from a *Malus *(apple) cDNA sequence suggesting that this gene may be transcriptionally merged with the neighboring squalene epoxidase-like gene (site 4b).

### Micro-colinearity with Arabidopsis

By cross-referencing Arabidopsis proteins identified by Blastx to their respective physical map positions, instances of microcolinearity or conserved microsynteny with *Arabidopsis thaliana *were identified in twelve fosmids, involving a total of 36 putative *Fragaria *genes (Table 3). As an example of microcolinearity in fosmid 08G19 (Figure 12), *Fragaria *genes 2 and 3 are homologous to Arabidopsis loci At3g54340 and At3g54350. As examples of interrupted colinearity but conserved microsynteny, *Fragaria *genes 4 and 6 in fosmid 76K13 (Figure 10) are homologous to Arabidopsis loci At1g21600 and At1g21610, while *Fragaria *genes 5 and 6 in fosmid 10B08 (Figure 11) are homologous to Arabidopsis loci At1g27880 and At1g27850.

### Transposable-Element-Related Sequences

Two full length COPIA-type LTR retrotransposons were identified: one on fosmid 14K06 (Figure 6 - site 2) and one on fosmid 19M24 (Figure 18). Single *Mutator*-like elements were identified on fosmids 10B08 (Figure 11) and 52B01 (Figure 15 - site 2). Gene sites 4 and 1 on fosmids 73I22 (Figure 2) and 76C08 (Figure 5), respectively, encoded putative reverse transcriptase-related proteins, while gene sites 1 and 5 on fosmids 14K06 (Figure 6) and 49M15 (Figure 21), respectively, encoded transposase-like proteins. Blastx searches detected retroviral polyprotein-like sequences on fosmids 08G19 (Figure 12 - site 7), 19H07 (Figure 16 - site 8), 53J04 (Figure 9 - site 7), and 53O08 (Figure 4 - site 4).

### SSR content

Any uninterrupted tract of five or more identical repeat units (of repeat length ≤ 5 nucleotides) was counted as an SSR by the SSRIT analysis. Part E of each Fosmid Figure depicts the locations, motif sequences, and repeat lengths of these SSR sites. The number of SSR sites per fosmid insert varied from zero (fosmid 32L07 - Figure 20) to 19 (fosmid 49M15 - Figure 21). The total number of qualifying SSRs found in the 20 targeted fosmid inserts was 158, giving an average SSR distribution of ~1 per 4.5 kb (Table 4). There were 123 di-nucleotide repeat SSRs and 35 tri-nucleotide repeats, and no tetra- or penta- nucleotide repeats. Of SSRs with repeat numbers of 18 or more, two were AT (= AT and TA) repeats and 13 were AG (= AG and CT) repeats. The eight longest SSR tracts were all AG repeats, ranging from 24 to 38 repeats. Among tri-nucleotide repeat types, AAG (= AAG, AGA, and GAA) repeats were by far the most common. The numbers/frequencies of the various SSR repeat types are summarized in Table 4.

## Discussion

Our evaluation of targeted gene space in the strawberry diploid species, *Fragaria vesca*, unveils new knowledge about 20 important genomic neighborhoods: information that can guide a diversity of gene- or trait-specific investigations, and facilitate site-specific molecular marker development. Moreover, the cumulative generation of over 1.75 Mb of genomic sequence by the present investigation of 20 gene-targeted sites and its companion study of 31 randomly selected sites [16], provides an invaluable baseline of robustly assembled and carefully annotated Sanger sequence data to which future Next Generation data sets and high throughput bioinformatic analyses can be compared and assessed. We anticipate that the experience gained through this effort will contribute valuable perspective, precedent, and impetus to whole genome sequencing efforts in *Fragaria*.

Although our assessment of gene content ultimately relied on homology-based methods, *ab initio *predictions provided an illuminating framework within which to organize and interpret homology-based determinations. In undertaking a comparison of the six higher plant *ab initio *training models (*Arabidopsis*, *Medicago*, monocot, *Nicotiana*, *Lycopersicon*, *Vitis*) accessible on Softberry's FGENESH website [23], we hypothesized that the taxa most closely related phylogenetically to *Fragaria *would provide the best training models for our analysis. According to the most recent release of the Angiosperm Phylogeny Group [24], the ordered phylogenetic distances of the six training model taxa from *Fragaria *(order Rosales) are (closest to most distant) *Medicago *(Fabales) <*Arabidopsis *(Brassicales) <*Vitis *(Vitales) <*Nicotiana *and *Lycopersicon *(Solonales) < monocots. By one measure, the "accurate" prediction of start and stop codons, the *Medicago *(Mt) model was marginally better than the *Arabidopsis *(At) model, and both of these surpassed the remaining four predictive models. It would be of considerable interest to know whether and to what extent an FGENESH model trained on Rosaceae sequence data would outperform the Mt and At models for predictive analysis of *Fragaria *sequence; however, such a model was not available for the present study.

Our analysis indicated that the six FGENESH models were variably prone to over-prediction, under-prediction, gene-merging, and/or gene-splitting. However, with knowledge of these tendencies in hand, the overall perspective provided by comparisons among these disparate models provided a useful backdrop to the interpretation of homology-based analyses, helping to draw attention to structural anomalies worthy of further exploration. On balance, our experience suggests that integrated consideration of all six FGENESH model outputs provided maximal insight into the genetic content of the studied *Fragaria *sequences. As easily visualized by viewing a broad sampling of the Fosmid Figures, the FGENESH models were in substantial agreement in some genomic regions, but at considerable variance in others. Yet such disagreements are themselves informative, potentially drawing attention to sites of unconventional functionality.

### Gene content

The genomic frequency of gene sites identified in our study of 20 gene-targeted fosmid clones is similar to that found in 31 randomly selected clones from the same genomic library [16]. Discounting 11 TE-related gene sites, the present study identified 120 protein encoding genes and pseudogenes within a total of ~708 kb, or an average of one gene site per 5.9 kb. Similarly, the companion study identified 182 gene sites in 1,035 kb, or one gene per 5.7 kb [16]. That an intentional focus on gene-rich, as opposed to randomly selected, genomic sites yielded similar protein-encoding gene densities is consistent with the finding that TE and other repetitive sequence content in the *F. vesca *genome is quite low [16], and that most of this genome is, in fact, gene rich.

A surprising finding in the present study was the number of targeted genes, as well as non-targeted genes, that were tandemly duplicated. Full length tandem gene duplications of targeted genes were seen in fosmids 14K06 (*ADH*), 73I22 (*CHS*), 41O22 (*TPS*), 76K13 (*PISTILLATA*), 19M24 (NBS-LRR resistance-like), and 34E24 (NBS-LRR resistance-like). Tandem or near-tandem duplications of genes or gene fragments not targeted by probes were also seen. Such duplications involved apparently truncated pseudogenes on fosmids 13I24 (*CIPK20 KINASE*), 32L07 (*SMC2*), and 41O22 (pentatricopeptide containing protein). On fosmid 08G19, two apparently full-length copies of a small basic intrinsic protein gene flanked a retroelement-like sequence. Although the clustering and neighboring duplication of disease resistance-like genes is a well-known phenomenon in plants [25], it is noteworthy that, excluding the resistance-like genes, none of the homologues to the tandemly duplicated *Fragaria *genes enumerated above were themselves tandemly duplicated in Arabidopsis.

EST support varied with respect to the members of tandem gene duplicates. Substantial EST support existed for both *CHS *copies. In contrast, EST support was lacking for *ADH*-1, but was sufficient to allow definition of a gene model for *ADH*-2. Only one of the two *Pistillata *copies had top-tier EST support. Similarly, only one of the two tandemly duplicated NBS-LLR resistance-like genes on fosmid 19M24 had any top-tier EST support, while no such EST support existed for either *TPS *copy on fosmid 41O22 or for the single copy on fosmid 53J04. The absence of EST support for one or both members of a tandemly duplicated gene pair might be the consequence of differential expression patterns, or might simply be attributable to sampling bias in the existing *Fragaria *EST database, wherein most of the currently available sequences are from whole seedlings of *Fragaria vesca *subjected to a handful of stressors. Alternately, absence of EST support might be indicative of mutational gene silencing, which is one of several possible evolutionary fates of duplicated genes [26]. Resolution of these possibilities awaits the much needed expansion of the *Fragaria *EST database to include a comprehensive diversity of tissue types, and representation of influence by a broad spectrum of environmental variables.

### Targeted genes

The complete elucidation of candidate gene sequences from strawberry opens many opportunities to now test functional predictions as they relate to plant productivity. Clearly the information identified from analysis of *LEAFY*, *SOC*, *PHYA*, *HY5 *and *CO *all may present a means to now translate information about flowering from Arabidopsis and other species to strawberry. Strawberry species exhibit a wide range of photoperiodic behaviors. These are of intense interest to breeders as photoperiod sensitivity strongly dictates the utility of a given cultivar.

Anthocyanin pigmentation is an important aspect of fruit color and quality, but also can be a factor in stress resistance and other physiological functions and environmental interactions throughout the plant [27]. The identified *CHS*, *CHI*, *DFR*, and *RAN *genes are likely to be factors in many aspects of anthocyanin pigment composition and spatiotemporal distribution. Along with the anthocyanin pathway gene products, terpene synthases play a demonstrated role in flavor and fragrance as aspects for fruit quality, also making them of interest to strawberry breeders.

Two other metabolic genes, *ADH *and *GBSSI*, were of interest because of their widespread usage in plants [28], and their specific recent usage in *Fragaria *[5,19,29], for phylogenetic analysis. The finding that the *ADH *gene is tandemly duplicated in *F. vesca*, and the differential EST support for its two gene copies, further extends the potential interest in *ADH *as a focal point for comparative evolutionary studies in *Fragaria*. The *GBSSI *gene sequence described herein is that of *GBSSI*-1, as distinct from the *GBSSI*-2 gene used in the phylogenetic analysis of *Fragaria *by Rousseau-Gueutin et al. [5]. The presence of at least two copies of the *GBSSI *gene is a general feature of the Rosaceae family [21].

Disease resistance genes are of central interest to plant breeders. Conserved segments of NBS-LRR resistance-like genes have been isolated from genomic DNA in many plant species, including strawberry [30], using degenerate primers targeted to conserved sites [31]. The NBS-LRR and LRR resistance-like gene sequences we present here are the first complete genomic disease-resistance like gene sequences to be reported in strawberry.

### Colinearity

As the number of sequenced genomes grows, various studies have examined gene arrangement between sequenced genomes in the interest of inferring evolutionary relationships. One recent study defined microsyntenic relationships by examining colinearity of *Prunus *(a close taxonomic neighbor of *Fragaria*), *Populus*, *Medicago *and *Arabidopsis*. A positive relationship was defined as a distance not less than 200 kb that contained four gene pairs [32]. Comparisons using this approach relating *Prunus *and *Arabidopsis *genomes indicated that microsynteny is not well-conserved between these species. In the present study gene-pair relationships were examined between the genes ordered in the fosmids and the known gene order in Arabidopsis. Not surprisingly, similar results were obtained to those in the *Prunus*-*Arabidopsis *comparisons. The data in Table 3 indicate that out of the set of 20 fosmid clones, only nine shared evidence of potential gene-pair relationships with Arabidopsis.

The data agree well with the conclusions of Jung et al. [32]. There are some clear special cases that should be considered carefully. The two adjacent genes on fosmid 34E24 are NBS-LRR genes. These are typically found as proximally located members of a multigene family, so it is not surprising that these would be detected as colinear in these analyses. Two fosmids contain strawberry terpene synthase genes, where Arabidopsis only has one. In both cases an immediate neighbor is an Arabidopsis gene, yet a gene found on different linkage groups. This finding indicates the possibility that the terpene synthase gene may have been a site for duplication in strawberry relative to a common ancestor, or perhaps a site of duplication within strawberry.

### EST support and coverage

The genomic sequence analyzed provides a means to test gene prediction against actual gene-coding sequence, best estimated by analysis of EST relationships. Of the total predicted genes on all fosmids, approximately half (78/148) maintain >85% identity with an EST in the Viridiplantae database. When compared against ESTs from the Rosaceae even fewer matches were obtained, and those were typically from *Malus*, *Rosa *and *Prunus *where significant EST resources exist. Of all of the sequences featuring EST cognates, only fourteen genes have sufficient EST support to provide complete delineation of exon/intron boundaries as a basis for gene modeling, while 76 gene sites had no top-tier *Fragaria *EST support. Exemplifying the latter case, support was lacking for fosmid 08G19 gene 3 (Figure 12) and fosmid 10B08 gene 1 (Figure 11). The first is annotated only as an embryo defective transcript and the second is *Leafy*. Both of these are examples where transcripts may be expected to be found in specialized tissues and/or developmental contexts. Therefore, it is not surprising that representative cDNA sequences do not appear in the public databases, wherein over 90% of sequences represent seedling transcripts in response to abiotic stress.

Taken together, these findings indicate the need for more *Fragaria *EST coverage, especially from specific tissues and developmental states. EST coverage from other diploid species, such as *Fragaria iinumae*, will be helpful in the development of subgenome-specific markers in the cultivated strawberry *Fragaria *×*ananassa*. The reciprocal condition also exists, where fosmid-based sequences have EST coverage, but it is either confined to the Rosaceae (no match in Viridiplantae) or possibly strawberry specific (no match beyond *Fragaria*). These uncharacterized expressed sequences are abundant in EST collections but were not identified in this study.

### SSR loci

The identification of SSR loci for use as potential molecular markers for linkage mapping, marker assisted selection, and diversity studies has received considerable attention in *Fragaria *[33,34]. A total of 158 SSRs of five or more homogeneous repeat tracts were identified. Of the di-nucleotide repeat motif types, AG and AT were by far the most common, as has also been reported in species as diverse as Arabidopsis and rice [35]. Among tri-nucleotide repeat types, AAG was the most common, eclipsing the frequency of any other tri-nucleotide type by a factor of at least 2.8. AAG is also the most common tri-nucleotide repeat motif in Arabidopsis, while CCG is the most common type in rice [35].

The utilized SSRIT program counts only uninterrupted repeat tracts as SSRs. Thus, a continuous sequence such as (TCC)_6_TCT(TCC)_5 _(as in fosmid 14K06, SSRs D and E) would be counted as two SSRs by SSRIT because the two TCC tracts are interrupted by a TCT. From the perspective of PCR primer pair design for SSR marker genotyping, this and several other instances of close-proximity SSR tracts would have to be treated as a single SSR locus, amplified by a single primer pair. Thus, the total number of operationally defined SSR loci detected in the fosmid inserts is somewhat less than the total number of 158 counted SSRs. If any pair of SSR tracts separated by less than 100 bp is counted as constituting a single operational SSR locus for purposes of molecular marker development, there are 144 discrete SSR loci, with a frequency of 1 SSR locus per 4.9 kb, or about 200 SSR loci per Mb.

The current *F. vesca *linkage map [8] has a total length of 424 cM. Given the 206 Mb size of the *F. vesca *genome, there is an average ratio of 486 kb/cM. Extrapolating from these data, SSR loci are distributed in the *F. vesca *genome with a density of about 92 SSR loci per 1 cM of map distance, thus indicating that sufficient SSR loci exist to support the construction of SSR-based linkage maps to a resolution of well under 1 cM.

### Repetitive elements

In this study, thirteen TE-related elements were detected on the basis of Blastx homology and structural analysis. A thorough analysis of TE-related and other repetitive element content in 31 random sequence samples comprising ~1 Mbp in *F. vesca *was presented in the companion study [16], while Ma et al. [36] reported the isolation of retroelement sequences from *Fragaria *×*ananassa*. No top-tier EST support was found for any of the TE-related sequences identified in the present study or that of Ma et al. [36], and no evidence of contemporary TE transpositional activity has been reported to date in *Fragaria*.

## Conclusions

Characterization of a targeted sampling of gene space in strawberry provides tremendous information that can be used on many levels. First, a comprehensive accounting of genic regions revealed by Sanger sequencing will inform and guide high throughput, whole genome sequencing efforts, and serve to anchor short-read sequencing assemblies. The focus on genes with known roles in biological processes relevant to agricultural production allows comparative study of these genes and their nascent transcripts, and development of markers for use in breeding and selection. The results allow contrast with other genomes, spotlighting the surprising tendency for gene duplications in strawberry. The detailed characterization of diploid strawberry gene space also is a reference point that will permit comparisons with other diploid and polyploid *Fragaria *species, further unveiling evolutionary relationships in this economically important genus.

## Methods

### Plant Material

*Fragaria vesca *ssp. *americana *variety 'Pawtuckaway' was collected from a site on Mt. Pawtuckaway in Deerfield, New Hampshire by T. M. Davis and S. Williamson, and was propagated and maintained in the Department of Biological Sciences Greenhouse facility at the University of New Hampshire (UNH). It has been donated to the National Clonal Germplasm Repository (NCGR) [37] in Corvallis, OR, wherein it is identified as accession CFRA 1948.001 (PI 657856).

### DNA Extraction

Total genomic DNA was isolated from two grams of freshly harvested, unexpanded leaves from greenhouse-grown plants using a modified 2% CTAB protocol [38], but without addition of 100 μl 24:1 chloroform:octanol to the tissue slurry after grinding in liquid nitrogen. Modifications also included the addition of antioxidants (0.10% (w/v) ascorbic acid/0.13% (w/v) sodium metabisulfite (Na_2_O_5_S_2_), added to CTAB just prior to use, followed by incubation on ice rather than at 60°C. No steps were taken to exclude organelle DNA.

### Fosmid Library Construction

Fosmid cloning was performed using the Epicentre CopyControl™ Fosmid Library Production Kit per the manufacturer's instructions (Epicentre, Madison, WI). End-repaired DNA (264 ng) was ligated into the CopyControl™ pCC1FOS™ fosmid vector (Epicentre), packaged, transfected into EPI300™-T1^R ^*E. coli *cells (Epicentre), and the transformed cells were stored in a 20% glycerol freezing buffer at -80°C. Fosmid clones were robotically picked into 384-well plates (Genetix, Boston, MA) and spotted in duplicate onto Performa II high-performance, positively charged, nylon high-density filters (Genetix) using a Genetix Qbot at the Hubbard Center for Genome Studies (HCGS), UNH. Clones were grown in a 4% glycerol enriched LB freezing buffer, and stored at -80°C.

### Probe Construction, Filter Hybridization, and Clone Selection

Labeled probes with an average size of 650 bp were synthesized by incorporation of Biotin-16-2'-deoxy-uridine-5'-triphosphate (Biotin-16-dUTP) (Roche, Indianapolis, IN) into PCR products. Primer pairs used in probe synthesis were designed using DNAStar LaserGene PrimerSelect Mac OS X 5.53 software, based on unpublished data generated in house, public domain *Fragaria *EST sequences, or conserved regions of orthologous genes. PCR reactions were prepared using MasterTaq kits (Eppendorf, Westbury, NY). Filters were hybridized using the Standard Hybridization protocol described in the NEBlot^® ^Phototope^® ^Kit (NEB, Ipswich, MA), with minor modifications. Probes were used in multiplexed sets of two to six. All clones to which protein-encoding, nuclear gene probes hybridized were subjected to confirmatory PCR screening using the same primer pairs used to generate the respective probes. When multiple clones were obtained for a single probe, clones were fingerprinted by restriction digestion, and insert ends were sequenced. Any clone in which the target gene was adjacent to and possibly truncated by the insert end was excluded from subsequent sequencing.

### Fosmid Subcloning and Sequencing

Fosmid clones that were selected on the basis of positive hybridization to probes and further differentiated on the basis of end-sequences and restriction digests were subcloned and sequenced as described in Pontaroli et al. [16]. Fosmid clones were sequenced to 14× redundancy, using an ABI 3700 capillary sequencer with T3 and T7 primers and ABI PRISM Big Dye Terminator chemistry (Applied BioSystems, Foster, CA). Base calling and quality assessment were done by using PHRED [39], and reads were assembled with PHRAP. Contigs were ordered using CONSED [40]. Assembled insert sequences were deposited into the GenBank database [41].

### Bioinformatics

For each fosmid, predictions of protein-encoding gene content, including start and stop codon predictions, were obtained using FGENESH (Softberry) [23], as trained on six reference models: *Arabidopsis thaliana *(At), *Medicago sativa *(Mt), Monocots (Mo), *Nicotiana tabacum *(Nt), *Lycopersicon esculentum *(Le), and *Vitis vinifera *(Vv). To obtain homology-based gene inferences, each fosmid sequence was subjected to Blastx and Blastn (NCBI) [41] queries of relevant GenBank databases using default parameters, except that "maximum target sequences" was set at 1000, and under formatting options, "graphical overview" was set at 1000 and the default low complexity filter was turned off. Blastx searches were done against the *Arabidopsis thaliana *and Viridiplantae databases, for the purposes of locating putative protein-encoding genes and assigning them to the plus or minus strand, inferring start and stop codon locations, and establishing putative gene identities. Blastn searches of the "EST others" database and limited to the Rosaceae family of plants were used to identify supporting ESTs in *Fragaria *and other rosaceous species as a means of further validating gene locations, inferring start/stop codon positions, and identifying expressed sites that lacked Blastx protein homology matches or FGENESH gene predictions. The locations of inferred start and stop codon sites were then compared to the start and stop codon locations predicted for the respective genes by each of the FGENESH models. Blastn and tBlastx searches of the Viridiplantae nr database were performed on a limited, *ad hoc *basis as needed to resolve questions that emerged from the systematic analyses. Gene models for genes with complete EST coverage were devised by integrating start and stop codon identifications with exon/intron boundary predictions generated independently using GeneSeqer [42].

The identification of transposable-element-like sequences employed a multifaceted approach. Full-length LTR retrotransposons were discovered by structural search using LTR_FINDER [43] and LTR_STRUC [44]. Novel repeat elements were identified using RepeatModeler [45]. Blastx was also used to discern TE-related protein-encoding sequences.

Simple sequence repeats (SSRs) were identified using SSRIT [46] with search parameters set for pentamers as the maximum repeat length and five as the minimum number of repeat units. The LaserGene ^® ^suite of programs (DNASTAR) was used for various purposes, including visualization and annotation of reading frame translations, construction of EST contigs, and sequence alignments.

## Authors' contributions

As project Principle Investigator, TMD conceived and co-managed the project, performed a substantial component of annotation, and drafted the manuscript. MES constructed the fosmid library, performed annotation, created the fosmid figures, and contributed substantially to manuscript preparation. QZ performed all probing and fosmid isolation procedures and contributed to annotation. DTT contributed to annotation. JLB supervised fosmid subcloning and sequence assembly, supervised structural analysis of TE content, and provided some manuscript revisions. ACP performed fosmid subcloning and contributed to annotation. HW and QY performed structural analysis of TE content. PSM performed the DNA sequencing and sequence assembly. KMF was project Co-Principal Investigator, contributed to project design and management, data analysis, and manuscript writing. All authors read and approved the final manuscript.

## Supplementary Material

Additional file 1 **Fosmid Gene Content Annotation**. An Excel spreadsheet of gene content of 20 sequenced fosmids as determined by homology searches. Click here for file

Additional file 2 **Gene Models**. An Excel spreadsheet of exon/intron composition of 15 modeled genes. Click here for file

## References

[B1] Ichijima K Cytological and genetic studies on *Fragaria* Genetics 1926 11 6 590 604 17246472 10.1093/genetics/11.6.590 PMC1200919

[B2] Hummer KE Nathewet P Yanagi T Decaploidy in *Fragaria iturupensis *Staudt (Rosaceae) Am J Bot 2008 96 3 713 716 10.3732/ajb.0800285 21628226

[B3] Davis TM Denoyes-Rothan B Lercerteau-Kohler E Kole C Strawberry Fruits and Nuts 2007 4 Berlin: Springer-Verlag 189 206 full_text

[B4] Folta KM Davis TM Strawberry genes and genomics Crit Rev Plant Sci 2006 25 5 399 415 10.1080/07352680600824831

[B5] Rousseau-Gueutin M Gaston A Aïnouche A Aïnouche ML Olbricht K Staudt G Richard L Denoyes-Rothan B Tracking the evolutionary history of polyploidy in *Fragaria *L. (strawberry): new insights from phylogenetic analyses of low-copy nuclear genes Mol Phylogenet Evol 2009 51 3 515 530 10.1016/j.ympev.2008.12.024 19166953

[B6] Shulaev V Korban SS Sosinski B Abbott AG Aldwinckle HS Folta KM Iezzoni A Main D Arus P Dandekar AM Multiple models for Rosaceae genomics Plant Physiol 2008 147 3 985 1003 10.1104/pp.107.115618 18487361 PMC2442536

[B7] Davis TM Yu H A linkage map of the diploid strawberry, *Fragaria vesca* J Hered 1997 88 3 215 221

[B8] Sargent DJ Clarke J Simpson DW Tobutt KR Arus P Monfort A Vilanova S Denoyes-Rothan B Rousseau M Folta KM An enhanced microsatellite map of diploid *Fragaria* Theor Appl Genet 2006 112 7 1349 1359 10.1007/s00122-006-0237-y 16505996

[B9] Oosumi T Gruszewski HA Blischak LA Baxter AJ Wadl PA Shuman JL Veilleux RE Shulaev V High-efficiency transformation of the diploid strawberry (*Fragaria vesca*) for functional genomics Planta 2006 223 6 1219 1230 10.1007/s00425-005-0170-3 16320068

[B10] Folta KM Davis TM Transformation systems to study gene function in *Fragaria* Proceedings 2007 North American Strawberry Symposium: February 9-12, 2007; Ventura, California 2007 North American Strawberry Growers Association, Kemptville, ON Canada 94 99

[B11] Slovin J HSP101 in the model strawberry *Fragaria vesca* HortScience 2005 40 4 1016 http://www.ars.usda.gov/research/publications/publications.htm?SEQ_NO_115=177284

[B12] Sosinski B Shulaev V Dhingra A Kalyanaraman A Bumgarner R Rokhsar D Verde A Velasco R Abbott AG Folta KM, Gardiner SE Rosaceous genome sequencing: Perspectives and progress Genetics and Genomics of Rosaceae 2009 New York, NY: Springer 601 615 full_text

[B13] Bevan M Bancroft I Bent E Love K Goodman H Dean C Bergkamp R Dirkse W Van Staveren M Stiekema W Analysis of 1.9 Mb of contiguous sequence from chromosome 4 of *Arabidopsis thaliana* Nature 1998 391 6666 485 488 10.1038/35140 9461215

[B14] Hoeven R Van der Ronning C Giovannoni J Martin G Tanksley S Deductions about the number, organization, and evolution of genes in the tomato genome based on analysis of a large expressed sequence tag collection and selective genomic sequencing Plant Cell 2002 14 7 1441 1456 10.1105/tpc.010478 12119366 PMC150698

[B15] Lai CWJ Yu QY Hou SB Skelton RL Jones MR Lewis KLT Murray J Eustice M Guan PZ Agbayani R Analysis of papaya BAC end sequences reveals first insights into the organization of a fruit tree genome Molecular Genetics and Genomics 2006 276 1 1 12 10.1007/s00438-006-0122-z 16703363

[B16] Pontaroli AC Rogers RL Zhang Q Shields ME Davis TM Folta KM SanMiguel P Bennetzen JL Gene content and distribution in the nuclear genome of *Fragaria vesca* The Plant Genome 2009 2 1 93 101 10.3835/plantgenome2008.09.0007

[B17] Shields ME Construction and characterization of a large-insert genomic library for *Fragaria *(Rosaceae) 2005 M.S. Durham, NH: University of New Hampshire

[B18] Davis TM Folta KM Shields ME Zhang Q Gene pair markers: An innovative tool for comparative linkage mapping North American Strawberry Symposium March 11, 2008 2007; Ventura, California 2007 North American Strawberry Growers Association, Kemptville, ON Canada 105 107 http://www.intl-pag.org/15/abstracts/PAG15_P03f_193.html

[B19] Staudt G DiMeglio LM Davis TM Gerstberger P *Fragaria *× *bifera *Duch.: Origin and taxonomy Bot Jahrb Syst 2003 125 1 53 72 10.1127/0006-8152/2003/0125-0053

[B20] Wolyn DJ Jelenkovic G Nucleotide sequence of an alcohol dehydrogenase gene in octoploid strawberry (*Fragaria *× *ananassa *Duch) Plant Mol Biol 1990 14 5 855 857 10.1007/BF00016518 2102862

[B21] Evans RC Alice LA Campbell CS Kellogg EA Dickinson TA The granule-bound starch synthase (GBSSI) gene in the Rosaceae: multiple loci and phylogenetic utility Mol Phylogenet Evol 2000 17 3 388 400 10.1006/mpev.2000.0828 11133193

[B22] Deng C Davis TM Molecular identification of the yellow fruit color (c) locus in diploid strawberry: a candidate gene approach Theor Appl Genet 2001 103 2-3 316 322 10.1007/s001220100648

[B23] Fgenesh HMM-based gene structure prediction http://www.softberry.com

[B24] A.P.G.III An update of the Angiosperm Phylogeny Group classification for the orders and families of flowering plants: APG III. The Angiosperm Phylogeny Group Botanical Journal of the Linnean Society 2009 161 105 121 10.1111/j.1095-8339.2009.00996.x

[B25] Friedman AR Baker BJ The evolution of resistance genes in multi-protein plant resistance systems Curr Opin Genet Dev 2007 17 6 493 499 10.1016/j.gde.2007.08.014 17942300

[B26] Moore RC Purugganan MD The evolutionary dynamics of plant duplicate genes Curr Opin Plant Biol 2005 8 2 122 128 10.1016/j.pbi.2004.12.001 15752990

[B27] Meyers BC Kaushik S Nandety RS Evolving disease resistance genes Curr Opin Plant Biol 2005 8 2 129 134 10.1016/j.pbi.2005.01.002 15752991

[B28] Small RL Cronn RC Wendel JF Use of nuclear genes for phylogeny reconstruction in plants Australian Systematic Botany 2004 17 2 145 170 10.1071/SB03015

[B29] Davis TM DiMeglio LM Identification of putative diploid genome donors to the octoploid cultivated strawberry, *Fragaria × ananassa* Plant & Animal Genomes XII Conference. San Diego, CA 2004

[B30] Martinez Zamora MG Castagnaro AP Diaz Ricci JC Isolation and diversity analysis of resistance gene analogues (RGAs) from cultivated and wild strawberries Mol Gen Genomics 2004 272 4 480 487 10.1007/s00438-004-1079-4 15565466

[B31] Leister D Ballvora A Salamini F Gebhardt C A PCR-based approach for isolating pathogen resistance genes from potato with potential for wide application in plants Nat Genet 1996 14 4 421 429 10.1038/ng1296-421 8944022

[B32] Jung S Jiwan D Cho I Lee T Abbott A Sosinski B Main D Synteny of *Prunus *and other model plant species BMC Genomics 2009 10 76 10.1186/1471-2164-10-76 19208249 PMC2647949

[B33] Davis TM DiMeglio LM Yang RH Styan SMN Lewers KS Assessment of SSR marker transfer from the cultivated strawberry to diploid strawberry species: Functionality, linkage group assignment, and use in diversity analysis J Am Soc Hortic Sci 2006 131 4 506 512 http://ddr.nal.usda.gov/bitstream/10113/21530/1/IND43832028.pdf

[B34] Sargent DJ Davis TM Simpson DW eds Strawberry (*Fragaria *spp.) structural genomics 2009 New York: Springer

[B35] Lawson MJ Zhang L Distinct patterns of SSR distribution in the Arabidopsis thaliana and rice genomes Genome Biol 2006 7 2 R14 10.1186/gb-2006-7-2-r14 16507170 PMC1431726

[B36] Ma Y Sun H Zhao G Dai H Gao X Li H Zhang Z Isolation and characterization of genomic retrotransposon sequences from octoploid strawberry (*Fragaria *× *ananassa *Duch.) Plant Cell Rep 2008 27 3 499 507 10.1007/s00299-007-0476-7 18026732

[B37] National Clonal Germplasm Repository http://www.ars.usda.gov

[B38] Davis TM Yu H Haigis KM Mcgowan PJ Template mixing - a method of enhancing detection and interpretation of codominant RAPD markers Theor Appl Genet 1995 91 4 582 588 10.1007/BF00223283 24169884

[B39] Ewing B Hillier L Wendl MC Green P Base-calling of automated sequencer traces using phred. I. Accuracy assessment Genome Res 1998 8 3 175 185 9521921 10.1101/gr.8.3.175

[B40] Gordon D Abajian C Green P Consed: A graphical tool for sequence finishing Genome Res 1998 8 3 195 202 9521923 10.1101/gr.8.3.195

[B41] National Center for Biotechnology Information http://www.ncbi.nlm.nih.gov/

[B42] Schlueter SD Dong Q Brendel V GeneSeqer@PlantGDB: Gene structure prediction in plant genomes Nucleic Acids Res 2003 31 13 3597 3600 10.1093/nar/gkg533 12824374 PMC168940

[B43] Xu Z Wang H LTR_FINDER: an efficient tool for the prediction of full-length LTR retrotransposons Nucleic Acids Res 2007 35 W265 W268 10.1093/nar/gkm286 17485477 PMC1933203

[B44] McCarthy EM McDonald JF LTR_STRUC: a novel search and identification program for LTR retrotransposons Bioinformatics 2003 19 3 362 367 10.1093/bioinformatics/btf878 12584121

[B45] RepeatModeler http://www.repeatmasker.org/RepeatModeler.html

[B46] Temnykh S Lukashova A Cartinhour S DeClerck G Lipovich L McCouch S Computational and experimental analysis of microsatellites in rice (*Oryza sativa *L.): frequency, length variation, transposon associations, and genetic marker potential Genome Res 2001 11 1441 1452 10.1101/gr.184001 11483586 PMC311097

